# Commercial plasma donation and individual health in impoverished rural China

**DOI:** 10.1186/s13561-014-0030-6

**Published:** 2014-11-18

**Authors:** Xi Chen

**Affiliations:** School of Public Health, Institution for Social and Policy Studies and Department of Economics, Yale University, New Haven, CT 06520 USA

**Keywords:** Paid plasma donation, Poverty, Panel data, HIV/AIDS, Hepatitis, Health status

## Abstract

**Background:**

Blood collection following nonstandard operations largely increases the risks of infectious diseases through cross-contamination. Commercial plasma donation and the resulting HIV/AIDS and hepatitis C epidemics in central China in the 1990s killed more than one million people. Many blood banks have since moved to more remote southwest provinces, which have become new suppliers of blood plasma.

**Methods:**

Utilizing a primary longitudinal survey, this paper documents commercial plasma donation and estimates its negative health impacts in impoverished rural China using individual fixed effect models. Both the linear regression model and generalized linear models are utilized.

**Results:**

Attracted by the financial compensation, a majority of plasma donors are poor, and bear grave consequences of malnutrition and worse health status as a result of unhygienic and frequent donations. Donating plasma is associated with a .83 standard deviation (SD) decline in self-rated health, a .54 SD lower self-rated health relative to peers in their age group, a .74 SD higher chance of being infected with hepatitis, lacking of strength to conduct farm work, and experiencing appetite loss, fatigue, nausea, and vomiting.

**Discussion:**

Results indicate an urgent need of more comprehensive and effective interventions on hepatitis screening, diagnosis, and treatment among plasma donors in less developed contexts to eliminate cross-infection of infectious diseases and possible widespread epidemic in the future. Besides, we should encourage voluntary plasma donation to gradually crowd out paid donation.

**Electronic supplementary material:**

The online version of this article (doi:10.1186/s13561-014-0030-6) contains supplementary material, which is available to authorized users.

## Background

Whether human bodies, including human blood, should be excluded from trade, has been a fundamental question under heated debate. Some argue that legalizing commercial blood donations may crowd out voluntary and selfless donors who signal pro-social characteristics, and financially incentivized donors would be more likely to transmit diseases than voluntary donors. Paid donations raise major concerns over quality risks (Titmuss, 1970; Zhao, 2007; WHO, 2014), partly due to asymmetric information in that blood product buyers are not in a position to know what they buy, whereas blood product suppliers know what they sell (Arrow 1972).

There are two separate blood markets, one for whole blood and the other for blood plasma. The former is mainly used for clinical blood transfusion, and the latter is extracted by pharmaceutical companies to produce blood products, such as intravenous immunoglobulin (IVIG), albumin, and anti-inhibitor. Some countries allow no extraction of blood plasma from whole blood. Many countries stipulate that whole blood donation must be on a voluntary basis with no financial compensation, while in some countries tiny compensation or gift is offered to cover transportation cost and nutrition subsidy. However, large financial compensations are often provided to encourage blood plasma donation on a regular basis, especially in underdeveloped areas (Kanbur, 2004).

Paid plasma donors are often labeled poor and rely on plasma donation as a source of income. Offering large financial incentives would cause those in need of money to take too many risks. First, frequent plasma donation without adequate nutrients compensation may greatly endanger health status. Second, the nonstandard operations to collect plasma are particularly worrisome in impoverished contexts, because they often ignore sterilization, lack accurate virus detection methods, improperly share centrifuge machines or use non-disposable needles (Prati, 2006). The high risk of infectious diseases through cross-contamination on plasmapheresis, such as hepatitis C, may lead to chronic liver disease, cirrhosis and even hepatocellular carcinoma (Alter, 2007). In the long run, malnutrition and infectious diseases may accumulate devastating impacts on the human capital of poor plasma donors and undermine governmental poverty alleviation efforts.

Meanwhile, understanding blood contamination and the epidemic of infectious diseases is important to the interests of various social entities. Besides donors trying to minimize the risks against own health, patients who demand blood products care about infectious diseases associated with plasma transfusion. Even the general public worry about epidemic of infectious diseases that may bear grave consequences. For instance, the induced HIV/AIDS crises in China’s Henan province in the 1990s caused thousands of deaths and left children unattended. Some living AIDS carriers have married, had children, or moved to other parts of China amid the largest internal migration process in human history. A rapidly growing epidemic of hepatitis attacked Guizhou in early 2006 during which the incidence, the number of deaths, and the fatality rate of the population in Guizhou all increased strikingly (China HIV/AIDS Information Network CHAIN 2006). Local blood banks were shut down shortly afterwards.

Further, the rapid economic growth in China and some other fast developing countries has been accompanied by strong domestic demand for blood products. The integration into the world economy has brought in large external demand for domestic blood products, which may exacerbate the concern for supply shortage and inferior blood quality.

However, in large part due to the difficulty in collecting individual level data on stigmatized paid plasma donation, there have been few empirical studies in China on this issue, especially after China’s major HIV/AIDS epidemic in Henan province in the 1990s and a major hepatitis epidemic in Guizhou province in the 2000s. This paper attempts to fill this gap. While the gradual implementation and enforcement of the *Blood Donation Law* in China has generally promoted screening and diagnosis of potential infectious diseases among plasma donors, the progress has been uneven. This paper provides such evidence from impoverished rural China, where most blood plasma donors reside. Since the poor react more to the large financial incentives for plasma donation and therefore are more vulnerable to health risks associated with plasma donation, we focus on evaluating their health impacts as a result of frequent plasma donations with nonstandard operations in unhygienic conditions.

To investigate health impacts at the individual level, we administered a multi-wave census-type survey to follow the same individuals over seven years. Information on self-reported health status, hepatitis infection, detailed household and individual socioeconomic conditions as well as community characteristics was collected from all individuals in 26 villages in rural western China. The longitudinal feature of the dataset allows us to estimate an individual fixed effect model to remove potential time-invariant confounded factors and draw more reliable conclusions on health impacts of frequent plasma donation in unhygienic conditions.

The main findings in this paper include: 1) the payoff from plasma donation, the pressure to get the son married, and the family age structure all drive up commercial plasma donation; 2) frequent plasma donation is associated with significantly worse self-rated health outcomes and a higher chance of being infected with hepatitis among the poor; 3) frequently donating plasma may affect donors’ ability to engage in farm work via feeling feeble, being easily infected with diseases, or experiencing physical discomfort in the busy season; 4) a majority of migrants, whether they suffered from work accidents or work-related disabilities, believe that plasma donation is riskier than industrial injuries.

The rest of this paper is organized as follows. In section 2, plasma donation in China is documented. Section 3 describes our multi-wave data collection process. Section 4 reports empirical results on the socioeconomic determinants of commercial plasma donation and provides evidence on its negative health impacts. Finally, section 5 concludes with a discussion about the policy and the limitation of the study.

## Methods

### Study context

In China, the markets for whole blood and blood plasma are separate: the former is mainly supplied by voluntary or employer-organized donations in urban area, while the latter prevails in rural areas and offers cash compensation to blood plasma donors. The current regulation forbids pharmaceutical companies extracting plasma from voluntarily donated whole blood. The greater commercial demand for blood plasma makes it much more popular than whole blood donations. Besides significant income increase for plasma donors and booming profit for pharmaceutical companies, the plasma economy becomes a lucrative source of income for middlemen.

The national regulation for plasma donation stipulates that individuals are excluded from giving if they are older than 50 or younger than 20, weigh less than 50 kgs for males and 45kgs for females, or seriously disabled. In addition, an interval of 15 days between donations is required for plasma, and 3 months for whole blood (Asia Catalyst, 2007). Once the plasma is separated from the whole blood, the red blood cells are re-injected back into the donor intravenously. To speed up the process, a donor can be given blood from previous donors with the same blood type and sent on his way while his blood is being processed in a centrifuge machine.

Though tremendous efforts have been made, tragedies of contamination among blood donors have left shadows on the safety of the plasma derivative industry (Zhu, 2007). For example, in some cases even people tested positive with hepatitis are allowed to give plasma; their plasma is simply placed in a different pile. Contamination of red blood cells during the process of obtaining plasma was strongly suggested by the high prevalence of hepatitis antibodies among repeat plasma donors. Compared to whole blood donation, the rate of hepatitis infection for plasma donation in China has been three times higher (Gao et al., 2011).

Outbreaks of HIV infection among commercial plasma donors from different areas occurred as early as 1994 (Wu et al., 2001). These have been attributable to the unhygienic conditions under which plasma donation has been carried out, with no proper sterilization procedure implemented. Consequently, blood plasma donors, together with injecting drug users (IDUs), have accounted for more than two thirds of China’s HIV infection, and as many as 250,000 blood plasma donors became infected by 2004 (Cohen, 2004). It is even estimated that by 2003, over 1.2 million people had contracted AIDS in Henan province alone, and blood transfusion in unhygienic blood banks is regarded as the root cause (Tsang, 2003; Gao, 2005). During the same period, the hemophilia patients infected by the use of contaminated blood or plasma products represent another set of severe social and medical problems.

There has also been a strong regional component to both paid blood plasma donations and the resultant outbreak of diseases, which has spurred government efforts to address this public health concern. For example, the widespread HIV/AIDS epidemic in China’s Henan province in the 1990s was attributed to unhygienic conditions for paid plasma donation. China rapidly responded to the epidemic by reducing the number of commercial blood banks and tightening regulations. Many blood banks in Henan province have since moved to more remote southwest provinces such as Guizhou, which has become a new supplier of blood plasma in China (Yin, 2006). Thus, the data for this study was collected mainly in that area.

In 2004, there were 23 blood plasma stations in Guizhou. By early 2006, they increased to 25 plasma stations, which accounted for 40 percent of the total blood plasma supply, rendering it the largest plasma market in the country. However, despite the efforts of the government to ensure greater attention to making blood plasma donations safe, in early 2006, a rapidly growing epidemic of infectious diseases hit Guizhou. As of January 2006, official data showed that the incidence, the number of deaths and the fatality rate of category B infectious disease increased by 21.36 percent, 65.38 percent and 36.28 percent on year-on-year basis respectively. By March 2006, the three numbers further increased to 30.01 percent, 73.17 percent and 33.20 percent, respectively (China HIV/AIDS Information Network CHAIN 2006). In the same year, all blood banks in Guizhou were shut down due to Hepatitis contamination.

After steps were taken by the government to improve the sanitary conditions of plasma donation in the region the blood banks in Guizhou were commercialized and re-opened in 2007. The regulation of the entire operation has been strengthened, through law and setting standards and upholding to stricter licensing regulations for plasma centers and fractionators. Public concerns over blood safety are gradually being relieved, and confidence is returning. However, this move switches blood bank ownership to pharmaceutical companies that produce blood plasma products, which weakens regulation under the Ministry of Health during possible public health crises in the future.

Measures taken by commercialized blood banks to aggressively increase plasma donations include almost doubling the cash “nutrition” subsidy per donation and awarding cash prizes to registered donors at the end of each year. In addition, donors are required to donate once every half month, with cash penalties assessed when registered plasma donors delay their donation. Due to the incentive scheme, there is almost no difference in donation frequency throughout each year: donors usually donate plasma every half month. Meanwhile, once a family starts to donate plasma, it rarely stops. One obvious reason is that plasma donation generates a great proportion of income that is non-substitutable, while another important reason is that regular donors often feel a lack of energy to do farm work and have to rely on plasma donation eventually.

### Survey design and data collection

The dataset for this study consists of a three-wave census-type household survey in 26 randomly selected villages in rural western China’s Guizhou province (Additional file 1: Appendix Figure S1). Guizhou is one of the poorest provinces in China, while per capita GDP and other main socioeconomic indicators in our surveyed villages are close to the median level in Guizhou (Additional file 1: Appendix Table S1). More than 20 ethnic groups live there, including Miao, Buyi, Gelao, and Yi that in total comprise about 20 percent of the population, and the major Han group accounts for the rest 80 percent of population. All 802 households in 2005, 833 households in 2007, and 872 households in 2010 were interviewed, respectively. Due to the census feature of our survey, the growing number of households surveyed over time reflects the natural increase of households in these 26 villages. All the participants in this study provided their written informed consent to participate in this study. All the data used in this study has been anonymized. The survey design, implementation, the result estimations of this study, and potential ethics issues were approved by the IRB for Human Participants in the Office of Research Integrity and Assurance at Cornell University. The Protocol ID number is 0905000392, and the approved project is entitled "Blood Donation, Status Seeking and Health Outcomes".

At the beginning of 2005, the first-wave census survey of all 802 households collected detailed community characteristics and individual socioeconomic information. Plasma donations were collected on each family member, including those who were working outside the county at the time of survey. Given the sensitivity of collecting data on blood plasma donation, we made great efforts to ensure the accuracy of these data, including training of local enumerators who were able to effectively interact with the residents in our sample villages. Moreover, the enumerators were trained to identify and probe into major discrepancies between income and expenditures, a strong indication that respondents concealed plasma donation as an income source. In 2004, donors received a cash “nutrition” subsidy valued at 80 CNY for the plasma contained in 580 cc of blood. 31.2 percent households surveyed engaged in plasma donation, and the compensation accounted for 20.9 percent of per capita income (Table 1).

**Table 1:** **Income, poverty, income inequality and plasma donation compensation (2004–2009)**

	**2004**	**2006**	**2009**
Main sources of income (percent)			
*Farming*	33.3	31.4	33.1
*Livestock*	8.1	6.8	6.9
*Local non-farm and self-employment*	24.0	30.0	23.8
*Remittance from migrants outside the county*	8.0	13.1	8.8
*Disaster relief, anti-poverty programs, deforestation subsidies*	2.8	2.0	5.4
*Gift income*	5.6	9.1	8.2
*Plasma donation income*	20.9	4.3	6.1
Average annual plasma donation compensation per capita (CNY)	294.1	78.6	174.9
Cash compensation (or nutrition subsidy) in CNY for plasma donation (per 580 cc)	80	80	150
# households without plasma donor	552	749	749
# households with one plasma donor	235	66	97
# households with two or more plasma donors	15	18	26
Per capita annual income (CNY)	1404.7	1817.3	2855.6
Income inequality (Gini)	43.1	48.2	55.2
Income inequality excluding plasma donation (Gini)	46.3	49.0	56.6
Income below poverty line of 892 CNY (%)	37.3	36.3	22.4
Income below poverty line of US $1.25 per day using 2005 PPP (%)	71.3	64.1	52.7

*Source: Author’s three wave Guizhou survey data.*

Notes: 1 USD = 6.2 CNY, *PPP* purchasing power parity. The 2005 PPP exchange rate is at the “China-rural” level. See http://iresearch.worldbank.org/PovcalNet/jsp/index.jsp. The Poverty lines for 2004–2009 are adjusted according to the published annual inflation rate in various issues of China Statistic Year Book, published by China’s National Bureau of Statistics. The poverty line of 892 CNY per year in terms of PPP equals US $0.61 per day.

A follow-up survey of the same households was administered in early 2007 and all 833 households were interviewed. All individual hepatitis infection, other chronic disease statuses, and self-rated health status were surveyed. Blood banks in Guizhou were shutdown in 2006 due to hepatitis contamination in some branches. A few local residents were still able to donate through traveling to other counties nearby after the shutdown of the local blood bank in 2006, as the timing of shutting down differs. However, the overall plasma donation intensity in 2006 was much lower.

In early 2010, the third wave follow-up survey was conducted for all 872 households. Similar to the 2007 survey, individual hepatitis infection and self-rated health status were surveyed. Out of a range between 1 and 5 (the healthiest status equals 1, the least healthy status equals 5), the average self-evaluated health status equals 2.59, and the average self-evaluated health status relative to peers equals 2.78; both are better than the median level. In our Guizhou survey, the hepatitis infection rate among plasma donors reduced slightly from 37.08 percent in 2006 to 34.69 percent in 2009. The decreasing trend is likely due to more effective control measures taken by local blood banks in screening hepatitis infection. The recent prevalence of hepatitis infection among plasma donors is similar to the 33.95% (95% CI: 29.80% to 38.17%) reported in a systematic review of 64 studies on hepatitis C infection among plasma donors in China (Gao et al., 2011). However, 7.32 percent of residents in the 26 surveyed villages are hepatitis infected, a rate much higher than the estimated 3.2 percent in the general population of China^a^.

Overall, the lack of reduction in hepatitis infection rate in rural Guizhou may involve the following potential reasons. One, those who are not infected may migrate more. Two, the older cohort generally have a higher probability of hepatitis infection than the younger cohort and are less likely to migrate. Three, paid plasma donors are usually driven by economic benefits, therefore poor economic conditions may make cash compensation attractive. Four, a high hepatitis infection rate in the community increases the chances of exposure.

Compared to previous waves, nutrition subsidies have nearly doubled (from 80 CNY to 150 CNY) for the same volume of blood plasma. While by our third wave data collection the proportion of donors recovered to 14.1 percent of households and 6.1 percent of income, it was still much smaller than the 2004 year level (Table 1).

Throughout the three waves, women are more likely to donate plasma. Villages with more local odd job opportunities have higher proportions of female donors. These patterns may reflect intra-household labor divisions to accommodate agricultural production or off-farm work, as the opportunity cost for men to engage in these activities are higher than for women.

## Results

### Socioeconomic determinants of paid plasma donation

Before reporting the results on the health impact of plasma donation, we estimate the decision to donate plasma on the potential covariates at the individual level, the household level, and the village level. Table 2 presents the results.

**Table 2:** **Socioeconomic determinants of engagement in commercial plasma donation**

	**R1**		**R2**		**R3**	
	**Whether donate**		**Donate volume**		**# household**	
	**(1 = donate)**		**(log)**		**Members donate**	
***Household characteristics***						
Per capita income (log)	−0.042***	(0.014)	−0.282***	(0.098)	−0.072***	(0.021)
Cadre and party membership status (dummy)	−0.05	(0.045)	−0.429	(0.289)	−0.111**	(0.056)
Household size	0.026**	(0.011)	0.134*	(0.076)	0.033**	(0.015)
Year of education	0.004	(0.003)	0.026	(0.023)	0.005	(0.004)
Ethnicity status (dummy)	−0.164***	(0.061)	−1.100***	(0.394)	−0.299***	(0.075)
Share of elderly	−0.062	(0.079)	−0.525	(0.581)	−0.139	(0.106)
Share of unmarried son	0.180**	(0.077)	1.234**	(0.517)	0.204**	(0.098)
Ratio of farm wage to plasma compensation	−0.183	(0.145)	−1.279	(0.979)	−0.277	(0.173)
Exposure to big diseases (dummy)	−0.051*	(0.030)	−0.328	(0.205)	−0.033	(0.043)
Exposure to livestock deaths (dummy)	−0.033	(0.030)	−0.21	(0.202)	−0.026	(0.044)
Exposure to family member deaths (dummy)	−0.007	(0.051)	−0.114	(0.341)	−0.087	(0.061)
***Village characteristics***						
Mean per capita income (log)	−0.044	(0.065)	−0.266	(0.444)	0.042	(0.099)
Mean year of education	0.008	(0.034)	0.046	(0.220)	−0.008	(0.042)
Mean ethnicity status	−0.122	(0.177)	−1.189	(1.189)	−0.125	(0.191)
Mean share of elderly	0.208	(0.452)	1.127	(3.176)	0.226	(0.566)
Mean share of unmarried son	−0.397	(0.290)	−2.814	(2.036)	−0.471	(0.431)
***Year and village fixed effects***	Yes		Yes		Yes	
N	2507		2507		2507	

*Notes:* Robust standard errors are in the parentheses. *Significant at 10%; **Significant at 5%; ***Significant at 1%.

While the plasma donation compensation is rather trivial to the rich, the amount of cash is still a strong incentive for impoverished farmers. An intensive donor can earn much higher compensation from plasma donation than from any other income generating activities. Specifically, the compensation per plasma giving at least doubles the amount of daily wage in the local labor market. The increase in the relative price between daily labor wage and per plasma giving seems to discourage plasma donation. Poorer families are more likely to engage in paid plasma donation, donate more volume, and involve more family members in donation.

Plasma donation has been a major income source that helps lift households out of poverty in the short term (Additional file 1: Appendix Figure S2) and reduces income inequality (Table 1). Additional file 1: Appendix Figure S2 shows that a greater proportion of households have income higher than the national poverty line when income from plasma donation is included. Table 1 indicates that income inequality, measured by Gini coefficient, reduces when income from plasma donation is counted in.

The local sex ratio is highly skewed due to son preference and intense male farm labor demand (Additional file 1: Appendix Table S1). Many surplus men of marriage age often trigger competitive tensions in the marriage market, so parents with an unmarried son have to signal via building fancy houses for marriage (Wei et al., 2012), investing in expensive durable goods, throwing a costly wedding reception (Brown et al., 2011). They also have to work harder and participate in riskier income generating activities (Wei and Zhang, 2011). Consistent with these findings, one interesting pattern reflecting an unbalanced marriage market pressure is that families with an unmarried son are more intensively engaged in paid plasma donation.

Moreover, village leaders and ethnic minorities are less likely to donate plasma. The latter is due to an ethnic culture against giving blood. Besides, more senior members in a family is associated with less plasma donation. Families with members exposed to major diseases are less likely to engage in plasma donation. These findings suggest that current blood quality regulations on donors’ age eligibility and physical strength are somewhat effective.

Besides OLS regressions that assume the error term to be normally distributed, we estimate Generalized Linear Model (GLM) that allows more flexible error term distributions and more generalizable specifications. In Additional file 1: Appendix Table S2, GLM estimations on three main plasma donation decisions, i.e., whether donate, donate volume and number of household members donate, are presented. The results are very similar to the main results presented in Table 2.

### Negative health effects of paid plasma donation

Widely documented as a root cause of blood contamination and the resulting high prevalence of Hepatitis and HIV infection among commercial donors (Wu et al., 2001; Cohen, 2004), blood banks in rural China has been mixing blood from several donors in the same centrifuge to cut the excessive cost in obtaining plasma. As a result, blood banks in the surveyed region were shut down due to an epidemic of infectious diseases. In this section, we attempt to establish a positive relationship between blood plasma donation and the potential risk of disease infection and other negative (and even deadly) health impacts.

Though information on HIV infection is too sensitive to collect, in the second and the third waves of survey self-rated health and hepatitis infection were collected at the individual level. Plasma donation in unhygienic conditions may affect health status in different ways, including making body weak and infectious diseases. Therefore, Table 3 and Table 4 test the two channels. Comparing donors with non-donors in 2009 among those who did not donate plasma in 2006, the formal group is of worse health status, of worse health status relative to peers in their age group, and experiences a more serious deterioration in health status over time (Table 3).

**Table 3:** **Summary statistics on self-rated health status and hepatitis infection**

	**Mean**	**SD**
*Non-donor in both 2006 and 2009*		
*Change* in absolute self-rating	0.144	1.070
*Change* in relative self-rating	0.274	1.099
*Change* in hepatitis infection rate	0	0
*Non-donor in 2006 but donor in 2009*		
*Change* in absolute self-rating	1.575	0.929
*Change* in relative self-rating	1.154	0.689
*Change* in hepatitis infection rate	0.533	0.516
*Donor in 2006 but non-donor in 2009*		
*Change* in absolute self-rating	0.200	0.837
*Change* in relative self-rating	0.182	0.603
*Change* in hepatitis infection rate	0.083	0.289
*Donor in both 2006 and 2009*		
*Change* in absolute self-rating	1.688	0.830
*Change* in relative self-rating	1.607	1.005
*Change* in hepatitis infection rate	0.016	0.126

*Notes:* Both absolute self-rating and relative self-rating of health status range from 1–5. 1 corresponds to the healthiest status, while 5 indicates the least healthy status. Relative self-rating evaluates health status relative to peers of similar age.

**Table 4:** **Blood plasma donation, individual self-rated health status and hepatitis rate (individual fixed effect estimations, 2006 and 2009)**

	**R1**	**R2**	**R3**
	***Change*** **in absolute self-rating**	***Change*** **in relative self-rating**	***Change*** **in hepatitis rate**
*Changes* in donation decisions	0.823***	0.518**	0.192***
	(0.258)	(0.227)	(0.021)
Control variables	Yes	Yes	Yes
N	2775	3098	3189
R2 within	0.079	0.109	0.085

*Notes:* Robust standard errors are in the parentheses. **Significant at 5%; ***Significant at 1%. Both absolute self-rating and relative self-rating of health status range from 1–5. 1 corresponds to the healthiest status, while 5 indicates the least healthy status. The set of control variables is the same as Table 2. The difference in sample sizes between two regressions is due to missing values in self-rated health status. Fortunately, those with missing values in self-rated health status are no different from other respondents with regard to the set of socioeconomic covariates.

In the statistical analysis, individual fixed effect models are utilized to remove unobserved time-invariant factors at the individual level that could confound the identification of paid plasma donation on health status. With two-period longitudinal data, the individual fixed effect model is equivalent to a difference model that estimates how changes in individual plasma donation decisions between 2006 and 2009 affect changes in individual health and hepatitis status after controlling for changes in a rich set of covariates in Table 2. The changes in self-rated health status tease out systematic report errors that may exist in self-evaluated outcomes.

Results show that plasma donation is associated with significantly lower self-rated health status. Specifically, donating plasma in 2009 (but not 2006) is associated with a .83 standard deviation decline in self-rated health, a .54 standard deviation lower self-rated health relative to peers in their age group, and a .74 standard deviation higher likelihood of being infected with hepatitis (Table 4).

It is possible that frequently donating plasma to a certain extent starts to affect health and increase the risk of hepatitis infection. However, to our best knowledge, there is no such objective threshold recommended by medical professionals. As a remedy, we redefine plasma donation to be 1 if this person donates above the median level among all plasma donors and 0 if this person donates below the median level. We re-estimate Table 4 using this higher threshold, the results demonstrate even stronger impact of plasma donation decisions on self-rated health status. The impact on hepatitis rate does not change much, suggesting that the impact on hepatitis infection works mostly at the extensive margin. In other words, the likelihood of hepatitis infection is associated more with whether to donate than with changes in donation intensity. ^b^ These results are available upon request.

Meanwhile, blood plasma donation may have a devastating impact on agricultural production. Besides lacking of strength due to insufficient nutrients compensation after frequent plasma donation, donors infected with hepatitis may experience, among others, appetite loss, fatigue, nausea, and vomiting. In the second wave, information on physical strength and farming was collected from 527 households before the local government intervened and stopped the collection of more information on commercial plasma donation. Among them, 75 households engaged in blood plasma donation. 64.0 percent of donors become weak in strength and are easily infected with diseases. 67.6 percent of donors feel feeble and experience physical discomfort in the busy season.

Information on risk awareness associated with plasma donation was also collected from migrants and plasma donors in the sample. Though migrants are vulnerable to work-related accidents as they conduct highly intensive work in manufacturing plants, 61.9 percent of them believe that plasma donation is riskier than industrial injury. Even for migrants who experienced work accidents and are disabled, 19 out of 32 still believe that plasma donation is riskier. Due to the sensitivity of the issue, only a limited number of plasma donors were asked whether they had any health checks before donation, 6 out of 15 donors answered no. As a result of nonstandard operations, such as neglecting sterilization, lacking accurate detection methods, improper use of non-disposable needles, or sharing unhygienic centrifuge machines in collecting plasma, cross infection can be very serious if any donor carries an infectious virus.

## Conclusions

This paper documents commercial plasma donation in impoverished rural China. Though trivial to the rich, the financial compensation offers a strong incentive to the poor. We find that the payoff from plasma donation relative to local wage job, the pressure to get the son married, and the family age structure all affect the decision to donate plasma. Meanwhile, frequent plasma donation is associated with a worse self-rated health status, higher rates of hepatitis, and a lack of strength to engage in farm work.

The results indicate an urgent need of more comprehensive and effective interventions on hepatitis screening, diagnosis, and treatment among plasma donors in less developed contexts, which are important to control the transmission of infectious diseases and possible widespread epidemic in the future. Specifically, the key to reduce the negative health impact, especially the incidence of hepatitis or HIV/AIDS infection among plasma donors, is to encourage truly voluntary plasma donation and gradually eliminate paid plasma donation, separate high-risk from the low-risk volunteers, and eliminate cross-infection completely when collecting plasma. In addition, a hepatitis C virus RNA screening strategy was planned to roll out in all Chinese blood banks shortly after the third wave survey in 2010, which, if strictly enforced, should contribute to the reduction of hepatitis C virus prevalence rate.

## Discussion

Some limitations remain to be addressed. First of all, all health outcomes are self-reported, which may suffer from measurement errors. For example, due to the stigma and sensitivity associated with hepatitis infection, people infected may be more motivated to underreport plasma donation and therefore underestimate its impact on hepatitis infection. Fortunately, people with bad self-rated health may not be incentivized to underreport plasma donation. Therefore, comparing results from health outcomes with different degrees of measurement errors may mitigate this concern. Second, since no blood sample collection was allowed in our surveyed region, we do not have accurate individual level information on blood types and hepatitis types. Fortunately, most studies so far have found no significant differences between blood types and the rate of hepatitis infection (Gao et al., 2011). Meanwhile, though we cannot distinguish different hepatitis types, according to village clinics most of the reported hepatitis cases in our sample is hepatitis C, which at the population level accounts for more than 70 percent of all post-transfusion hepatitis infection. Third, compared to population level datasets, our survey is small in sample size, preventing us from investigating less frequently seen blood-borne diseases. Lastly, while individual fixed effect models are estimated and time-invariant confounding factors are removed, potential time-variant confounded factors may still exist.

## Endnotes

^a^The national infection rate and the infection rate in Guizhou may not be totally comparable as the national rate only includes hepatitis C infection, while the Guizhou survey did not separate hepatitis C infection from other types of hepatitis infection.

^b^Meanwhile, it is unlikely that there exists a universal threshold for all individual plasma donors over which donation is considered excessive. Rather, the threshold may vary by individual preference or health endowment. For example, a healthy donor may have a higher threshold than an ill donor. Our individual fixed effect estimation strategy using the longitudinal data attempts to remove individual level time-invariant preference and health endowment that may affect the decision to donate and the health impact. In other words, we estimate the impact of donation on health status given individual preference, endowment, etc.

## Additional file

Additional file 1: Appendix Figure S1. Location of the surveyed region. **Figure S2.** Distribution of Per Capita Income (2004). **Table S1.** Summary Statistics (2009). **Table S2.** Socioeconomic Determinants of Engagement in Commercial Plasma Donation (Results using Generalized Linear Models).
