# Supplementary material for: Commercial plasma donation and individual health in impoverished rural China
Source: Health Econ Rev. 2014 Nov 18;4:30. doi: 10.1186/s13561-014-0030-6 (PMC4502079; doi:10.1186/s13561-014-0030-6)
Supplement: Additional file 1: Appendix Figure S1. — Location of the surveyed region. Figure S2. Distribution of Per Capita Income (2004). Table S1. Summary Statistics (2009). Table S2. Socioeconomic Determinants of Engagement in Commercial Plasma Donation (Results using Generalized Linear Models). [file 13561_2014_30_MOESM1_ESM.docx]

**Appendix Figure 1 Location of the surveyed region**


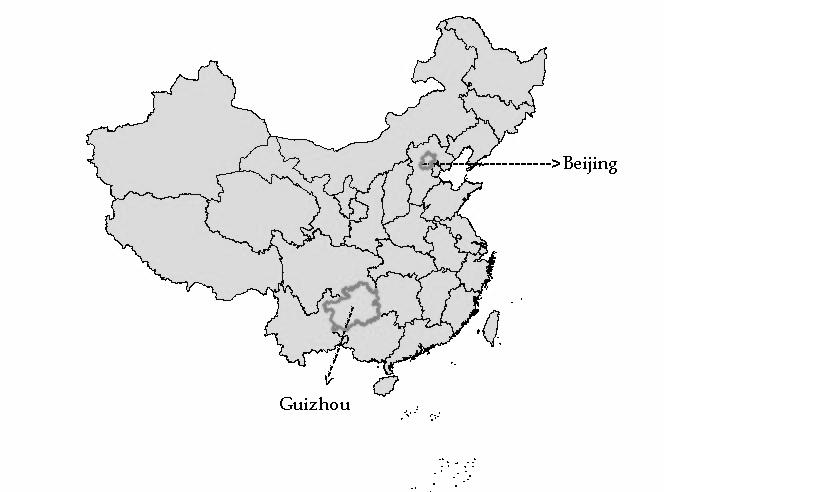

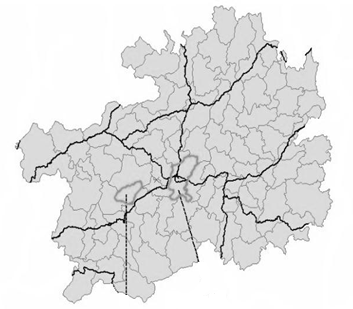


*Source:* Michigan China Data Center

**Appendix Figure 2 Distribution of Per Capita Income (2004)**

*Source: Authors’ 2004 survey data*

Note: 1 USD = 6.2 CNY

Two vertical lines, “L” and “H”, refer to the low (668 CNY) and high (892 CNY) official poverty lines stipulated by the Chinese government. Clearly, commercial blood plasma donation significantly moves the income distribution rightward with some households lifted out of poverty.

**Appendix Table 1 Summary Statistics (2009)**

|  | Mean | SD |
| --- | --- | --- |
| Number of villages | 26 | - |
| Distance to county seat (km) | 6.8 | 3.45 |
| Number of households | 872 | - |
| Total population | 3932 | - |
| Dummy for minority households | 35.15 | 0.46 |
| Dummy for male head of household | 92.82 | 0.26 |
| Education of household head (years) | 3.73 | 3.10 |
| Per capita cultivated land (mu) | 0.89 | 1.02 |
| Dummy for households with TV | 0.75 | 0.43 |
| Dummy for households with bicycles or motorcycles | 0.46 | 0.50 |
| Dummy for households with phones | 0.42 | 0.49 |
| Dummy for having difficulty with access to drinking water | 0.54 | 0.50 |
| Dummy for households with local odd jobs | 0.31 | 0.46 |
| Dummy for households with self-employment | 0.08 | 0.26 |
| Sex ratio for age cohorts 0-19 in 2004 | 1.13 | 0.17 |
| Sex ratio for age cohorts 0-19 in 2006 | 1.17 | 0.20 |
| Sex ratio for age cohorts 0-19 in 2009 | 1.16 | 0.18 |
| self-rated health status | 2.59 | 0.99 |
| self-rated health status relative to peers | 2.78 | 0.96 |
| Hepatitis infection rate (percent) | 7.32 | 26.04 |
| hepatitis infection rate among plasma donors (percent) | 34.69 | 47.84 |

*Source: Authors’ 3^rd^ wave Guizhou survey data*

*Notes:* Both absolute self-rating and relative self-rating of health status range from 1-5. 1 corresponds to the healthiest status, while 5 points to the least healthy status. Relative self-rating evaluates health status relative to peers of similar age.

**Appendix Table 2 Socioeconomic Determinants of Engagement in Commercial Plasma Donation**

**(Results using Generalized Linear Models)**

|  | R1 | | R2 | | R3 | |
| --- | --- | --- | --- | --- | --- | --- |
|  | Whether Donate  (1 = donate) | | Donate volume  (log) | | # household  members donate | |
| ***Household Characteristics*** |  | |  | |  | |
| Per capita income (log) | .733*** | (0.045) | -0.282*** | (0.098) | -0.085* | (0.047) |
| Cadre and party membership status (dummy) | 0.916 | (0.284) | -0.429 | (0.289) | -0.853** | (0.425) |
| Household size | 1.067 | (0.052) | 0.134* | (0.076) | 0.233*** | (0.045) |
| Year of education | 0.980 | (0.027) | 0.026 | (0.023) | -0.022 | (0.029) |
| Ethnicity status (dummy) | 0.839 | (0.400) | -1.100*** | (0.394) | -0.408 | (0.252) |
| Share of elderly | 0.449** | (0.151) | -0.525 | (0.581) | -3.039*** | (0.658) |
| Share of unmarried son | 4.683*** | (1.692) | 1.234** | (0.517) | 1.225*** | (0.305) |
| Ratio of farm wage to plasma compensation | 0.779 | (0.923) | -1.279 | (0.979) | -0.271 | (0.694) |
| Exposure to big diseases (dummy) | 0.947 | (0.443) | -0.328 | (0.205) | -0.059 | (0.330) |
| Exposure to livestock deaths (dummy) | 0.874 | (0.201) | -0.21 | (0.202) | -0.086 | (0.192) |
| Exposure to family member deaths (dummy) | 0.824 | (0.309) | -0.114 | (0.341) | -0.094 | (0.223) |
| ***Village Characteristics*** |  |  |  |  |  |  |
| Mean per capita income (log) | 0.641 | (0.318) | -0.266 | (0.444) | -0.023 | (0.362) |
| Mean year of education | 0.751 | (0.165) | 0.046 | (0.220) | 0.253 | (0.282) |
| Mean ethnicity status | 0.040* | (0.072) | -1.189 | (1.189) | -0.224 | (1.546) |
| Mean share of elderly | 1.093 | (2.779) | 1.127 | (3.176) | 3.184 | (2.091) |
| Mean share of unmarried son | 1.656 | (0.408) | -2.814 | (2.036) | 1.011 | (2.654) |
| ***Year and Village Fixed Effects*** | Yes | | Yes | | Yes | |
| N | 2507 | | 2507 | | 2507 | |

*Notes:* Robust standard errors are in the parentheses. *significant at 10%; **significant at 5%; ***significant at 1%. This table presents results in Table 2 using GLM estimations. Column R1 shows odds ratio, column R3 shows poisson regression coefficients.
